# Identification of genes and pathways potentially related to PHF20 by gene expression profile analysis of glioblastoma U87 cell line

**DOI:** 10.1186/s12935-017-0459-x

**Published:** 2017-10-04

**Authors:** Tianlong Liu, Tiejun Zhang, Feng Zhou, Jitao Wang, Xiaohu Zhai, Nan Mu, Jongsun Park, Minna Liu, Wenxing Liu, Peijin Shang, Yi Ding, Aidong Wen, Yuwen Li

**Affiliations:** 1 Department of Pharmacy, Xijing Hospital, Fourth Military Medical University, Xi’an, China; 2 Department of Pharmacy, The First Affiliated Hospital of SooChow University, Suzhou, China; 3 State Key Laboratory of Cancer Biology, Department of Biopharmaceutics, School of Pharmacy, Fourth Military Medical University, Xi’an, China; 4 Department of Pharmacology, Chungnam National University, Daejon, South Korea; 5 Department of Nephrology, Xijing Hospital, Fourth Military Medical University, Xi’an, China; 6 Department of Neurosurgery, The First Affiliated Hospital of SooChow University, Suzhou, China

**Keywords:** PHF20, Glioblastoma, U87 cell, Gene expression profile, Bioinformatics

## Abstract

**Background:**

Glioblastoma is the most common and aggressive brain tumor associated with a poor prognosis. Plant homeodomain finger protein 20 (PHF20) is highly expressed in primary human gliomas and its expression is associated with tumor grade. However, the molecular mechanism by which PHF20 regulates glioblastoma remains poorly understood.

**Methods:**

Genome wide gene expression analysis was performed to identify differentially expressed genes (DEGs) in U87 cells with PHF20 gene knockdown. Gene ontology (GO) and pathway enrichment analyses were performed to investigate the functions and pathways of DEGs. Pathway-net and signal-net analyses were conducted to identify the key genes and pathways related to PHF20.

**Results:**

Expression of 540 genes, including FEN1 and CCL3, were significantly altered upon PHF20 gene silencing. GO analysis results showed that DEGs were significantly enriched in small molecule metabolic and apoptotic processes. Pathway analysis indicated that DEGs were mainly involved in cancer and metabolic pathways. The MAPK, apoptosis and p53 signaling pathways were identified as the hub pathways in the pathway network, while PLCB1, NRAS and PIK3 s were hub genes in the signaling network.

**Conclusions:**

Our findings indicated that PHF20 is a pivotal upstream regulator. It affects the occurrence and development of glioma by regulating a series of tumor-related genes, such as FEN1, CCL3, PLCB1, NRAS and PIK3s, and activation of apoptosis signaling pathways. Therefore, PHF20 might be a novel biomarker for early diagnosis, and a potential target for glioblastoma therapies.

**Electronic supplementary material:**

The online version of this article (doi:10.1186/s12935-017-0459-x) contains supplementary material, which is available to authorized users.

## Background

Glioblastoma is the most common and lethal tumor of the central nervous system [1], owing to poor prognosis and repercussions on cognitive function [2]. Despite advances in knowledge and therapies over several decades, survival has not significantly improved, only 5.1% of patients with glioblastoma have a 5-year survival rate [3]. Thus, understanding the mechanisms that regulate glioblastoma progression is critical to developing novel therapies to improve patient outcome.

One particular protein of interest in glioblastoma regulation is plant homeodomain finger protein 20 (PHF20). PHF20 is a potent transcriptional activator, which binds to methylated lysine residues on the histone tail [4]. PHF20 is overexpressed in various cancer tissues compared to adjunct normal tissues, including advanced small-cell lung cancers and advanced adenocarcinomas [5]. Besides, PHF20 is highly expressed in primary human glioma specimens [6], and functions as an immunogenic antigen in glioblastoma [7, 8]. Auto-antibodies against PHF-20 were also detected in hepatocellular carcinoma [9] and meduloblastoma [10]. PHF20 expression levels have also been associated with the pathological tumor grade of gliomas [6].

To elucidate the mechanisms regulated by PHF20 in glioma as well as identify potential prognostic biomarkers and targets for drug discovery and immunotherapy, a microarray analysis was conducted to harness the systematic gene expression profile related to genomic and phenotypic information on glioblastoma in U87 cells.

## Methods

### Cell culture

Human glioblastoma cell lines U87, U251 and A172 originated from the Type Culture Collection of the Chinese Academy of Sciences (Shanghai, China). Cell lines LN229, HS683 and HEB were kindly provided by the department of neurosurgery at The First Affiliated Hospital of SooChow University. The cells were cultured in Dulbecco’s modified Eagle’s medium (DMEM) (Corning, NY, USA) containing 10% fetal bovine serum (FBS), 50 U/mL penicillin and 50 μg/mL streptomycin at 37 °C with 5% CO_2_ incubator. The cell lines tested negative for any mycoplasma contamination.

### Western blotting

1 × 10^6^ cultured cells were lysed with lysis buffer as previously described [5]. Protein concentration was measured using the BCA protein assay kit (Beyotime, Shanghai, China). The same amount of protein was separated by 10% sodium dodecyl sulfate–polyacrylamide (SDS-PAGE). A polyvinylidene difluoride (PVDF) membrane (Millipore, Bedford, MA, USA) was then used for electro-transfer. The membrane was blocked with 5% nonfat milk at room temperature for 1 h and incubated in primary antibodies against PHF20 (1:500, Cell Signaling Technology, USA), overnight at 4 °C. Subsequently, the membrane was incubated in the appropriate secondary antibody at room temperature for 1 h. In addition, β-actin was used as the loading control. Protein bands were visualized through enhanced chemiluminescence (ECL) reagent and detected using BioImaging Systems (UVP, Upland, CA, USA). The relative protein levels were calculated with Image J software (National Institutes of Health, USA). All experiments were performed in triplicate.

### Lentivirus-based shRNA infection

GFP-Lentiviral particles with PHF20-specific shRNA (shPHF20) were purchased from Genechem Co., Ltd. (Shanghai, China). The target sequence was TGACTTGGTTGTATCAGAT. Random sequence, TTCTCCGAACGTGTCACGT, was used as a negative control (shCON). U87 cells in 6-well plates were infected with lentiviral particles containing either shCON or shPHF20 to generate negative control (NC) or PHF20 knockdown (KD) U87 cells, respectively. 12 h after infection, the virus containing culture medium was replaced with fresh DMEM supplemented with 10% FBS for 72 h. The lentiviral infection efficiency was demonstrated by observing the presence of green fluorescent protein within the U87 cells using Olympus-IX71 fluorescence microscope (Tokyo, Japan) and RT-PCR assay.

### RNA extraction and quantitation

Total RNA was isolated using Trizol Reagent (Pufei, Shanghai, China) according to the manufacturer’s protocol. The RNA content was examined by identifying A260 and A280 values by using the Nanodrop 2000 (Thremo Scientific, Waltham, MA, USA). RNA integrity was assessed using a 2100 Bioanalyzer (Agilent Technologies) and an RNA 6000 Nano Kit (Agilent Technologies).

RNA with A260/A280 nm values over than 1.9, concentrations over 300 ng/μL and 28S/18S ratios over than 1.4 were used.

### Quantitative real time PCR analysis

Total RNA isolated was processed for cDNA synthesis using M-MLV reverse transcriptase (Promega Corporation, Madison, WI, USA). cDNA was amplified by PCR in StratageneMX3000p (Agilent Technologies, Santa Clara, CA, USA) using SYBR Master Mixture (TaKaRa, Tokyo, Japan). The expression levels of target genes were standardized against the GAPDH, an internal control, and calculated using the 2^−△△Ct^ method. The sequences of the primers used in PCRs are listed in Additional file 1. All the assays were performed in triplicate.

### mRNA microarray

Total RNA was processed for double-strand cDNA synthesis, IVT and amplified RNA fragmentation using the GeneChip 3′IVT Express Kit (Affymetrix, Santa Clara, CA, USA) according to the manufacturer’s instruction. RNA was then processed for hybridization at 45 °C for 17 h using The PrimeView™ Human Gene Expression Array (Affymetrix), which contains 49,395 probes covering more than 36,000 transcripts and variants. The arrays were washed in the GeneChip Fluidic Station 450 (Affymetrix), and scanned by the GeneChip Scanner 3000 (Affymetrix). These microarray data have been deposited in NCBI Gene Expression Omnibus (GEO) under accession number GSE93680.

### Data processing

The raw data, expressed as CEL files, were normalized by the log scale robust multi-array analysis (RMA) method with the Expression Console software version 1.1 (Affymetrix). The screening standard for a distinctly significant gene was an absolute fold change (|FC|) > 2 and a corrected p < 0.05.

### Gene ontology and pathway analysis

Gene ontology (GO) analysis was applied to analyze the main function of differentially expression genes (DEGs) according to the gene ontology, the key functional classification of National Center of Biotechnology Information (NCBI) [11]. Two-side Fisher’s exact test and χ^2^ tests were used to classify the GO category. The false discovery rate (FDR) [12] was calculated to correct the p value. The standard of difference screening was FDR < 0.05.

Pathway analysis was used to find out the significant pathway of the DEGs according to Kyoto Encyclopedia of Genes and Genomes (KEGG) [13]. The data analysis method and filter criteria were similar to the GO analysis.

### Pathway-net analysis

Pathway-net analysis was built according to the interaction among pathways of the KEGG database to directly and systemically determine the interaction among the significant pathways [14].

### Signaling processes analysis

Based on the KEGG pathway map (http://www.genome.jp/kegg/pathway.html) [15] ,DEGs involved in key pathways were labeled to clearly visualize the position of specific genes in the signaling processes and determine the regulatory role of DEGs involved in key pathways.

### Signal-net analysis

A gene–gene interaction network was constructed using the source of the interaction database from KEGG. For instance, if there is confirmative evidence that two genes interact with each other, an interaction edge is assigned between the two genes. The networks are stored and presented as graphs, where nodes represent main genes (protein, compound, etc.) and edges represent the relationship between the nodes, such as activation or phosphorylation. The algorithms and construction of the network were achieved using published methods [16].

## Results

### PHF20 is highly expressed in glioma cell lines

**Fig. 1:**
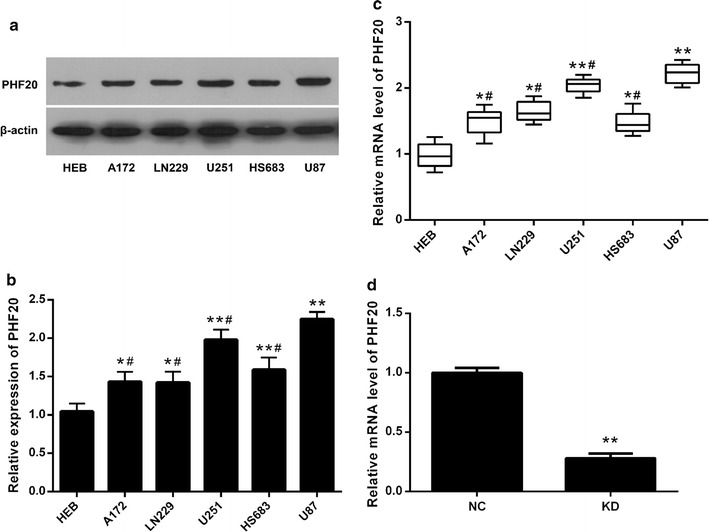
Expression of PHF20 in glioma cell lines (A172, LN229, U251, HS683 and U87) and human astrocytes cell line (HEB). **a** Expression of PHF20 was determined by immunoblotting (n = 3). **b** Relative protein levels were shown by density photometry. Error bars: ± SD. *p < 0.05, **p < 0.01 gliomas cell lines vs. HEB cell line; ^#^p < 0.05 A172, LN229, U251 and HS683 cell lines vs. U87 cell line. **c** Boxplots for the mRNA analysis by RT-PCR (n = 6). *p < 0.05, **p < 0.01 gliomas cell lines vs. HEB cell line; ^#^p < 0.05 A172, LN229, U251 and HS683 cell lines vs. U87 cell line. **d** mRNA levels of PHF20 were detected in U87 cells using qPCR following infection with shCON and shPHF20 (n = 3). Data presented as mean ± SD, **p < 0.01 KD group vs. NC group

We first examined the expression of PHF20 in various glioma cell lines. Expression of PHF20 protein was significantly higher in glioma cell lines (A172, LN229, U251, HS683 and U87) than that in human astrocyte cell line HEB (*p* < 0.05, Fig. 1a). The relative expression level of PHF20 in U87 cells was higher than in other glioma cell lines (*p* < 0.05, Fig. 1b). PHF20 mRNA level was verified by qPCR (Fig. 1c). Thus, the U87 cell line was used to establish PHF20 knockdown cells in following studies. The U87 cells were successfully infected with shCON and shPHF20 72 h after transfection, resulting in a 72% decrease in PHF20 expression (Fig. 1d).

### Identification of DEGs that regulated by PHF20

**Fig. 2:**
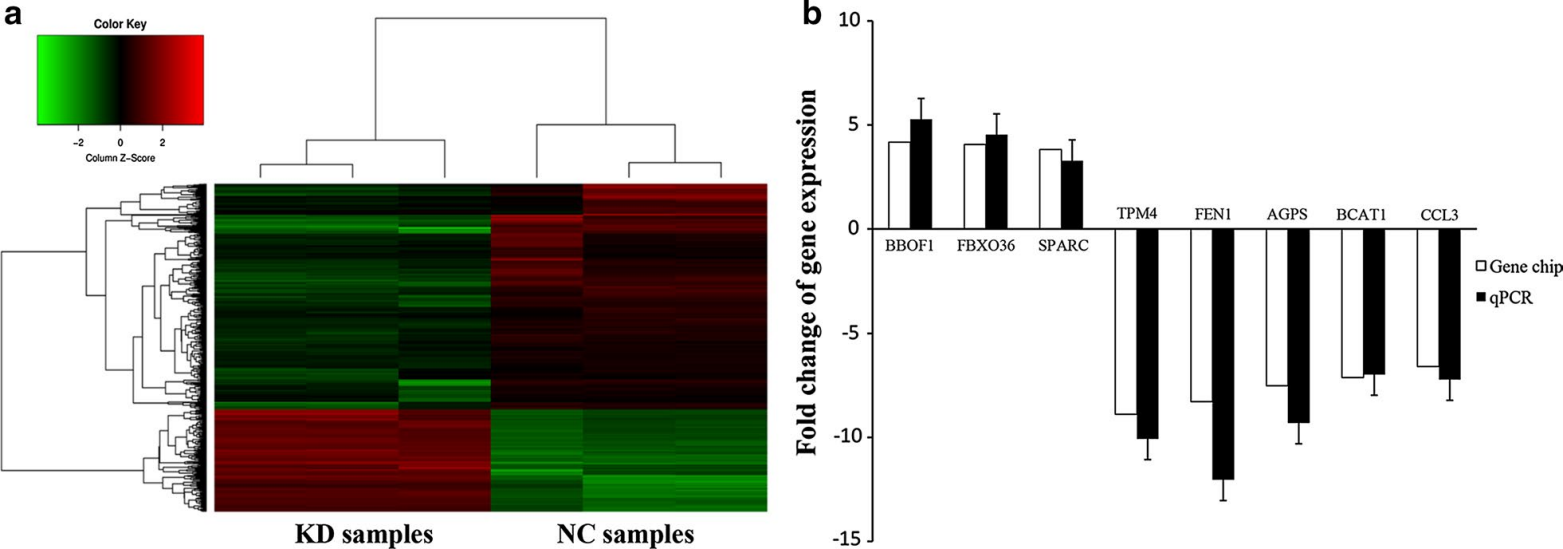
Differentially expressed genes (DEGs) between the U87 cells infected with the negative control (NC) and those infected with shPHF20 (KD). **a** Hierarchical clustering for DEGs. Green represents down-regulated genes, red represent up-regulated genes (p<0.05). **b** A subset of genes differentially expressed between NC and KD cells were validated by qPCR. White bars represent the fold change in expression level between KD and NC as indicated by microarray analysis. Black bars represent the mean fold change of gene expression calculated by qPCR method. GAPDH was used for normalization. Each grey bar is the mean ± SEM of three independent biological replicates

A genome wide gene expression analysis was carried out to identify DEGs between NC and KD U87 cells. A total of 540 DEGs were identified, including 175 up-regulated genes and 365 down-regulated genes (Fig. 2a, Additional file 2). A subset of DEGs was verified by qPCR. Expression of BBOF1, FBXO36 and SPARC increased, while the expression of TPM4, FEN1, AGPS, BCAT1 and CCL3 decreased in PHF20 knockdown cells (Fig. 2b).

### GO analysis of PHF20 associated DEGs

**Fig. 3:**
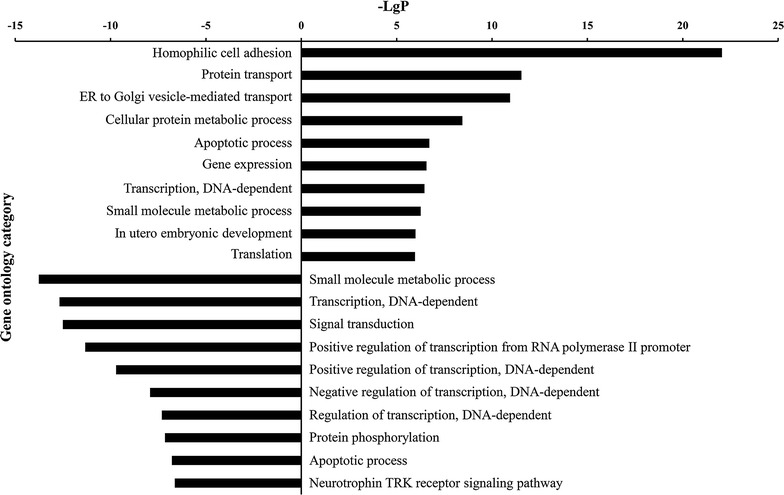
Histogram of gene ontology (GO) analysis of up-regulated and down-regulated genes upon PHF20 knockdown. The X axis displays the negative logarithm of the P value (− LgP) (the larger the value, the smaller the P value). The Y axis displays the name of the gene ontology category

To determine the primary functions regulated by PHF20 in glioma cells, a comprehensive gene ontology (GO) analysis was performed. A total of 540 DEGs were assigned to 236 GO terms (111 were up-regulated and 125 were down-regulated, Additional file 3). The analysis revealed that the up-regulated genes were significantly involved in homophilic cell adhesion, protein transport, and ER to Golgi vesicle-mediated transport. Down-regulated genes were involved in small molecule metabolic process, transcription (DNA-dependent), signal and transduction (Fig. 3). Additionally, both up and down-regulated genes were enriched in small molecule metabolic, transcription and apoptotic processes.

### KEGG pathway analysis of PHF20 associated DEGs

**Fig. 4:**
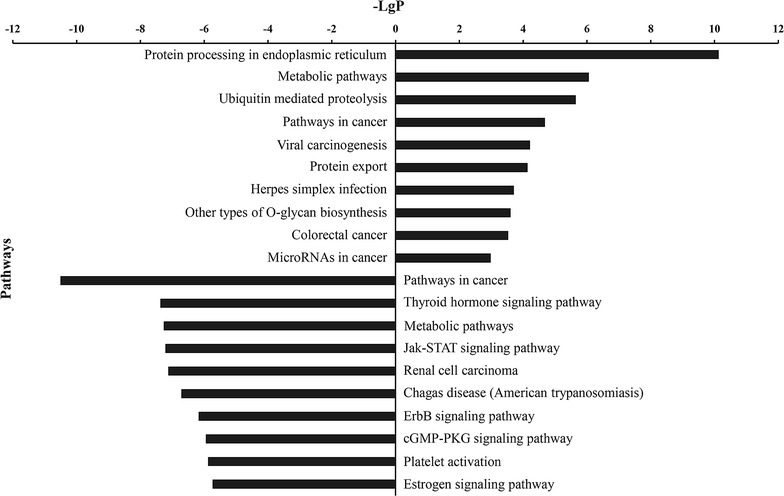
Histogram of significant signal pathways of up-regulated and down-regulated genes following PHF20 knockdown. The X axis displays the negative logarithm of the p value (− LgP) (the larger the value, the smaller the p value). The Y axis displayed the name of the pathway

Pathway enrichment analysis of DEGs was conducted on the basis of the KEGG pathway database. This analysis yielded 147 significant pathways, including 41 up-regulated pathways and 106 down-regulated pathways (see Additional file 4). The most significant up-regulated and down-regulated pathways were shown in Fig. 4. In addition, pathways in cancer were identified as up-regulated pathways as well as down-regulated pathways.

### Pathway-net analysis of significant PHF20-regulated pathways

**Fig. 5:**
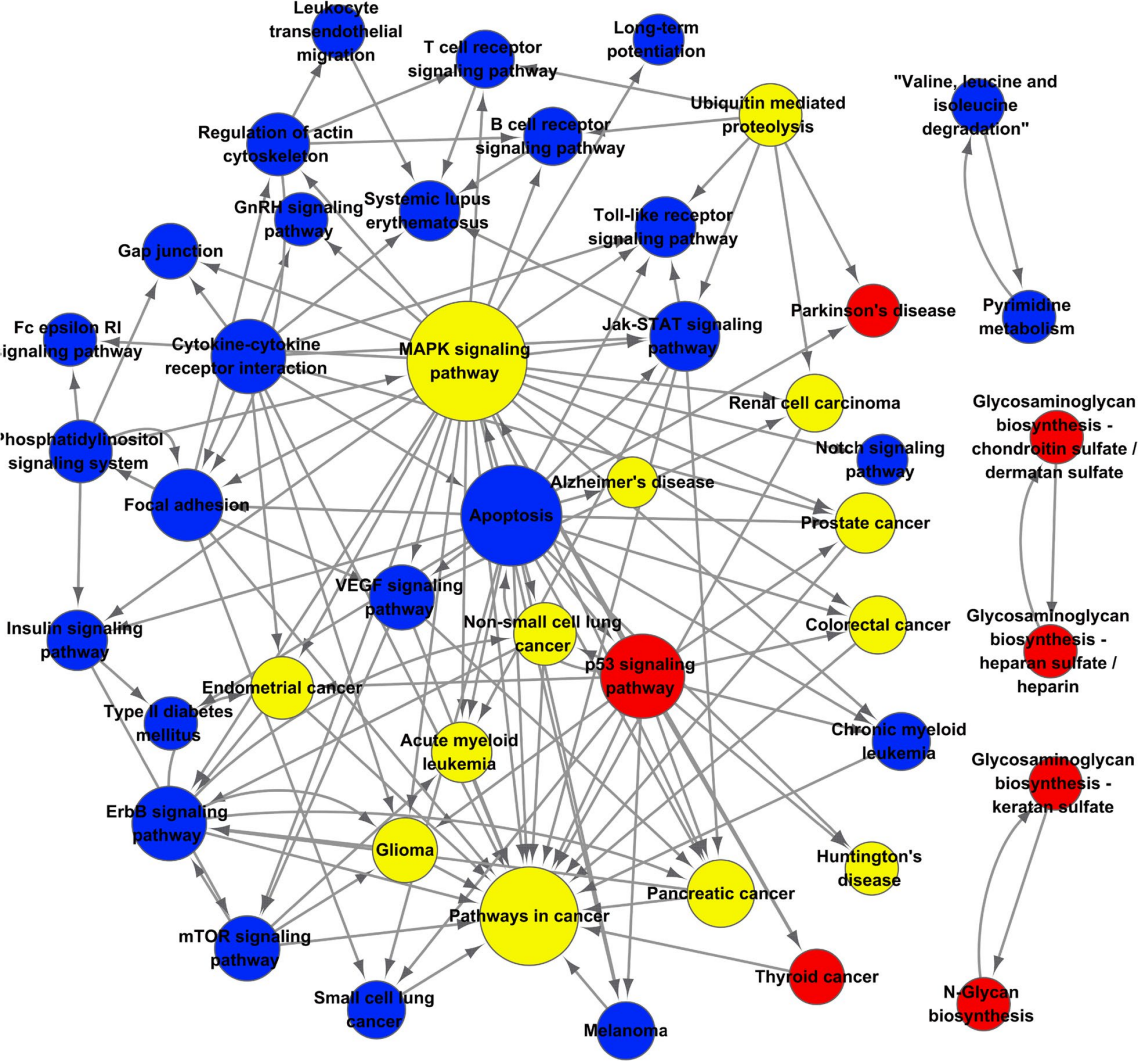
Representation of the interactions between significant pathways associated with PHF20 (pathway-net). The nodes represent pathways. The area of the nodes displayed represents the number of other pathways that interact with this pathway. Lines indicate interactions between pathways. The regulating pathway is represented by the arrow tail, while the pathway being regulated is represented by the arrowhead. Red nodes represent up-regulated pathways. Blue nodes represent down-regulated pathways. Yellow nodes represent the up/down-regulated pathways

In order to define functional relationships among pathways, an interaction net of the significant pathways associated with PHF20 was built (Fig. 5). 46 key pathways and the 147 connections between them were represented by nodes and edges, respectively. Primary interactions occurred between the MAPK, apoptosis, cancer, p53, ErbB, cytokine–cytokine receptor interaction, focal adhesion, and JAK-STAT signaling pathways (Table 1).

**Table 1:** The top 12 key pathways according to the degree size in pathway-net

Pathway name	Style	Outdegree^a^	Indegree^b^	Degree^c^
MAPK signaling pathway	Up, down	27	3	30
Apoptosis	Down	20	2	22
Pathways in cancer	Up, down	0	21	21
p53 signaling pathway	Up	14	1	15
ErbB signaling pathway	Down	5	6	11
Cytokine–cytokine receptor interaction	Down	11	0	11
Focal adhesion	Down	5	5	10
Jak-STAT signaling pathway	Down	5	4	9
Pancreatic cancer	Up, down	2	6	8
mTOR signaling pathway	Down	4	3	7
Glioma	Up, down	2	5	7
VEGF signaling pathway	Down	4	3	7

^a^Outdegree indicates the down-stream pathway numbers

^b^Indegree indicates the up-stream pathway numbers

^c^Degree indicates the sum of outdegree and indegree

### Signaling processes analysis of key pathways

**Fig. 6:**
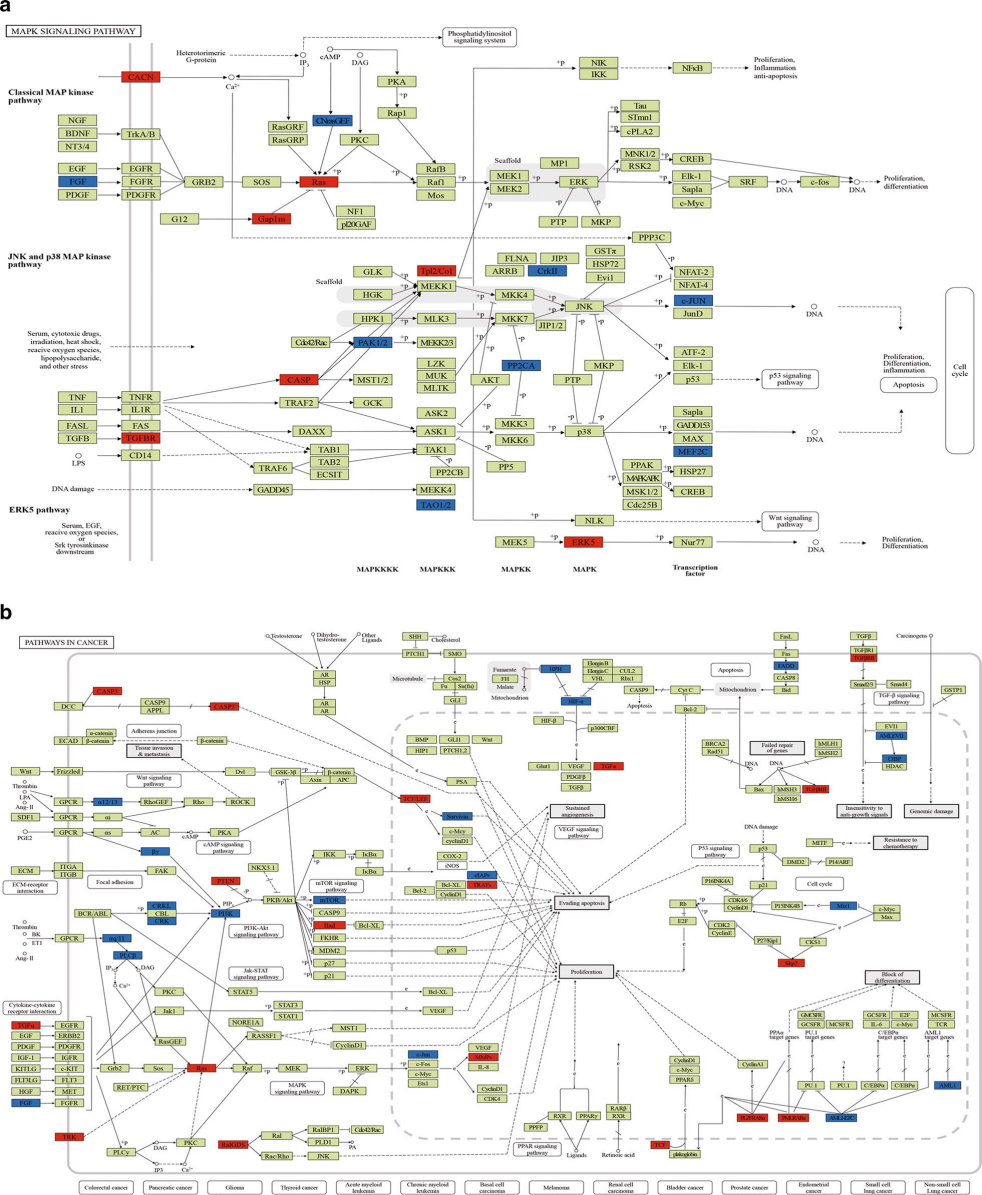
The regulatory roles of differentially expressed genes involved in the MAPK signaling pathway (**a**, map04010) and pathways in cancer (**b**, map05200). Red labels represent up-regulated genes; blue labels represent down-regulated genes. The metabolic maps of the signaling cascade demonstrate potential points regulated by PHF20. The original images were downloaded from the KEGG website

Signaling processes analysis, with the KEGG pathway map, was performed using DEG expression data to determine the regulatory role of DEGs involving in key pathways. As shown in Fig. 6, the MAPK signaling pathway included 7 up-regulated and 8 down-regulated genes while pathways in cancer contained 13 up-regulated and 20 down-regulated PHF20-regulated DEGs.

### Signal-net analysis of DEGs

**Fig. 7:**
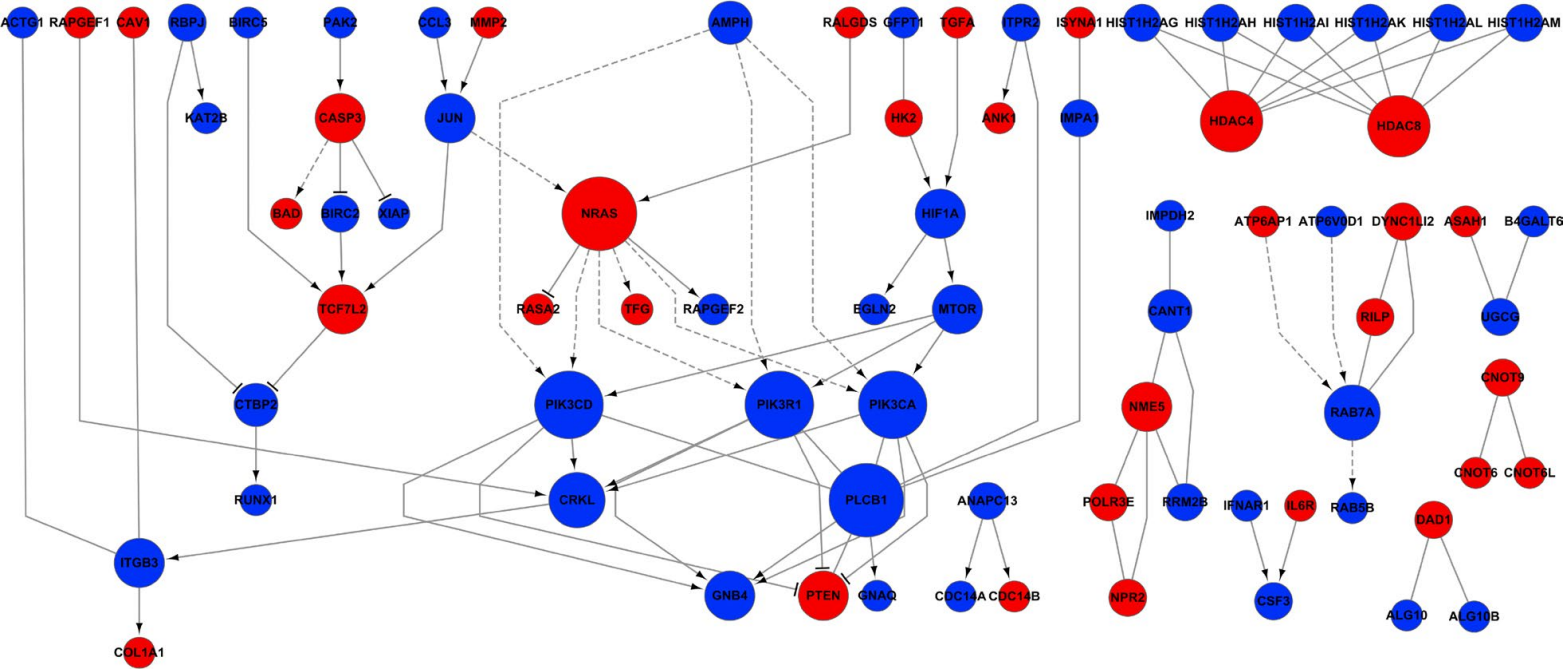
Interaction network analysis of differentially expressed genes related to PHF20. All differential gene interactions were analyzed in signal-net. The genes were connected in a network based on prior known protein–protein interactions and signaling pathways. Nodes represent genes. The area of the node represents the number of other genes that interact with the gene of interest. Lines indicate interactions between genes. Directed lines denote interactions, where genes being acted upon are indicated by the arrowhead and the regulating genes or upstream genes indicated at the arrow tail

Finally, we identified the key gene interactions between PHF20-related DEGs to construct a regulatory network map. 78 genes were included in the signaling network, and 106 potential direct interactions were identified (Fig. 7). PLCB1, NRAS, PIK3CD, PIK3CA, PIK3R1, HDAC4, HDAC8, CRKL, RAB7A and ITGB3 were the most significantly expressed genes according to the degree size (Table 2).

**Table 2:** The top 10 significantly expressed genes according to the degree size in signal-net

Gene symbol	Gene ID	Style	Outdegree^a^	Indegree^b^	Degree^c^
PLCB1	23236	Down	3	5	8
NRAS	4893	Up	6	2	8
PIK3CD	5293	Down	4	3	7
PIK3CA	5290	Down	4	3	7
PIK3R1	5295	Down	4	3	7
HDAC4	9759	Up	0	6	6
HDAC8	55869	Up	0	6	6
CRKL	1399	Down	1	4	5
RAB7A	7879	Down	1	4	5
ITGB3	3690	Down	1	3	4

^a^Outdegree indicates the down-stream gene numbers

^b^Indegree indicates the up-stream gene numbers

^c^Degree indicates the sum of outdegree and indegree

## Discussion

PHF20 was originally identified in glioma patients [8] and is significantly associated with glioma pathological tumor grade [6]. In recent years, a growing number of studies have shown that PHF20 is closely related to the development of various tumors [17, 18] and plays important roles in tumor suppression and progression. However, the underlying molecular pathways regulated by PHF20 in glioma remain largely undetermined. Therefore, further in-depth investigations are essential for better understanding of the biological roles of PHF20 in cancer.

In the present study, gene expression profile analysis was performed to identify differentially expressed genes (DEGs) between PHF20 knockdown U87 cells and negative control cells. A total of 540 genes (175 up-regulated genes and 365 down-regulated genes) were differentially expressed following knockdown of PHF20, which suggests that PHF20 may be a key regulator in glioblastoma. Multiple DEGs, including FEN1, BCAT1, AGPS and CCL3, have been implicated in the progression of various cancers. For example, FEN1 is overexpressed in glioblastoma [19]. FEN1 polymorphisms and variant genotypes are associated with glioma susceptibility [20, 21]. CCL3 is also highly expressed in glioma, and may promote glioblastoma cell proliferation and migration [22].

Gene ontology enrichment analysis revealed that highly enriched biological functions were related to PHF20, such as hemophilic cell adhesion, protein transport, metabolic process, transcription and apoptotic process. Thus, PHF20 may influence glioma progression by altering these biological processes.

Several DEG enriched pathways associated with tumorigensis were identified including protein processing in endoplasmic reticulum, metabolic pathways, ubiquitin mediated proteolysis, pathways in cancer, and thyroid hormone signaling pathways. Furthermore, pathway-net analysis revealed that multiple pathways participate in the occurrence and development of cancer including the p53 signaling pathway, apoptosis, pathways in cancer, and the TLR signaling pathway. The p53 signaling pathway was also enriched as a significant pathway by an array comparative genomic hybridization analysis in pilocytic astrocytoma [23]. Furthermore, our findings are in line with previous studies that found that PHF20 could stabilize and activate p53 by promoting p53 methylation [24], and that PHF20 inhibits p53 transcriptional activity via PKB mediated PHF20 phosphorylation [5]. A recent study also showed that PHF20 inhibits tumorigenicity by inducing apoptosis mediated by p53 and Bax [17]. Moreover, accumulating evidences suggested that PHF20 was expressed in a number of tumors, including glioma [6], lung cancer [25] and myeloid malignancies [26]. In addition, elevated expression of PHF20 could cause constitutive NF-B activation [6], which is a key downstream gene of TLR signaling pathway [27].

Finally, signal-net analysis revealed the interactions between 78 PHF20-regulated genes. Core genes PLCB1, PIK3CD/CA/R1, CRKL, RAB7A and ITGB3 were down-regulated while NRAS and HDAC4/8 were up-regulated. PLCB1 plays critical roles in intracellular transduction and regulating signal activation [28], which are important to tumorigenesis. As one of the RAS oncogene family, NRAS have been reported to be involved in development of leukemia [29], melanoma [30] and glioma [31, 32]. Members of the PIK3 family are frequently detected in a wide range of cancers and have been proposed as biomarkers for patient survival and drug response [33, 34]. PHF20 has been suggested as a substrate of PKB [5]. HDACs regulate various nuclear and cytoplasmic processes [35], which are common in various human neoplasms [36, 37]. In addition, synergistic anti-tumor actions between HDAC and PIK3 inhibitors have been validated [38].

## Conclusions

Overall, this study indicated that PHF20 is a pivotal upstream gene that influences the occurrence and development of glioma by regulating a series of tumor-related genes, like FEN1, CCL3, PLCB1, NRAS and PIK3s, and involved in apoptosis signaling pathways. Thus, PHF20 might be a novel biomarker for early diagnosis and therapeutic target for treatment of glioma. Nevertheless, further studies in molecular pathogenesis and large scale clinical tumor specimen validation are still needed.

## Additional files

**Additional file 1.** Primers used in real-time RT-PCR.

**Additional file 2.** The differentially expressed genes (DEGs) between common U87 cells and PHF20 knockdown U87 cells.

**Additional file 3.** Significant gene ontology (GO) analysis of differentially expressed genes (DEGs) related to PHF20.

**Additional file 4.** Significant pathway analysis of differentially expressed genes (DEGs) related to PHF20.

## Abbreviations

DEGs: differentially expressed genes
FDR: false discovery rate
GLEA2: glioma-expressed antigen 2
GO: gene ontology
HCA58: hepatocellular carcinoma associated antigen 58
KEGG: Kyoto Encyclopedia of Genes and Genomes
PHF20: plant homeodomain finger protein 20
