# Supplementary material for: Identification of genes and pathways potentially related to PHF20 by gene expression profile analysis of glioblastoma U87 cell line
Source: Cancer Cell Int. 2017 Oct 4;17:87. doi: 10.1186/s12935-017-0459-x (PMC5628484; doi:10.1186/s12935-017-0459-x)
Supplement: Supplementary file 1 — Additional file 1. Primers used in real-time RT-PCR. [file 12935_2017_459_MOESM1_ESM.docx]

Additional file 1. Primers used in real-time RT-PCR.

| Gene | Forward primer (5'-3') | Reverse primer (5'-3') |
| --- | --- | --- |
| PHF20 | TAGCTCCTACTGCTGTGGATT | AAGCCGAGGACGTTTTAATGG |
| TPM4 | AGCTCCGTGGGTCTTTCTCT | GAAAAAGCTGGTGCCATTTG |
| FEN1 | CGGGCTGTGGACCTCATC | CAAGTCGCCGCACGAT |
| AGPS | ACCAGATTCCCTGGAGTTCA | GAACCACCAGGTCCTCGATA |
| BCAT1 | TTCAACTCGTGATACACCAA | ATTCCTGTGCTAGAGAGCAT |
| CCL3 | TGCAACCAGTTCTCTGCATC | TGGCTGCTCGTCTCAAAGTA |
| BBOF1 | CCGTCGAAGGGAAAGGACAA | TCTGTGACTTCCAACCTGGC |
| FBXO36 | AGTTACTGGTCACCCGGTCT | AGGCAGTTTGACCTTGAAGATG |
| SPARC | TCGGCATCAAGCAGAAGGATA | ATTGGGGGAAACACGAAGGG |
| GAPDH | CAGCCTCAAGATCATCAGCA | TGTGGTCATGAGTCCTTCCA |
